# Pyelitis in Pregnancy

**Published:** 1908-07

**Authors:** F. G. Hodgson

**Affiliations:** Gynaecologist to Wesley Memorial Hospital, Surgeon to Presbyterian Hospital, Gynaecologist and Abdominal Surgeon to Tabernacle Infirmary, Instructor in Gynaecology in Atlanta College of Physicians and Surgeons, Atlanta, Ga.


### PYELITIS IN PREGNANCY.*

BY F. G. HODGSON, M. D., GYNAECOLOGIST TO WESLEY MEMORIAL HOSPITAL, SURGEON TO PRESBYTERIAN HOSPITAL, GYNAECOLOGIST AND ABDOMINAL SURGEON TO TABERNACLE INFIRMARY, INSTRUCTOR IN GYNAECOLOGY IN ATLANTA COLLEGE OF PHYSICIANS AND SURGEONS, ATLANTA, GA.

This may be defined as an inflammation of the pelvis of the kidney occurring during pregnancy and may be continued into the puerperium.

Attention was first called to this condition by Chamberlain of this country in the American Journal of Obstetrics for April, 1877. He said that the enlarged uterus caused retention of urine in the kidney, and subsequent inflammation of the pelvis of the kidney. Kruse, of Wurzburg, next drew attention to this condition in 1899. At the French Congress of Surgeons in 1892, Reblaub reported five cases. Vinay and Bonneau in 1893 first noted that the kidney of the right side was the more frequently affected. Dr. Navas, of Lyons, in 1897, asserted that the cause of the inflammation was usually the colon bacillus. Vinay, in 1898, at the Congress of Obstetrics and Gynaecology of Marseilles, re-

*Read before Fulton County Medical Society, March 5, 1908.

ported eight additional cases and noted that cases may occur as early as the second month of pregnancy; that the cause is usually the b. coli communis; and that the prognosis is generally good. Weill, in 1899, said that the infection usually took place through the blood and not by ascending from the bladder. Cragin, in New York has made a careful study of these cases and has reported twenty-three cases in his practice. Many other observers have reported cases and the literature of the subject is quite extensive.

The ureters are subject to the pressure of the enlarged uterus, especially at the point where they enter the brim of the true pelvis. Here the uterus presses them up against the resistant bony wall of the pelvis and dams up the urine in the ureters and pelvis of the kidneys. (In a number of autopsies upon pregnant or recently delivered women the ureters and pelves of the kidneys have been found in an irregularly dilated condition.) The pressure upon the ureters causes a stasis of urine or uronephrosis. This in trun causes a congestion of the ureter and pelvis of of the kidney. So we have a very favorable condition for the development of inflammation. It has been found that this inflammation developes more frequently on the right side. This is because of the oblique position of the uterus due to the rectum being on the left side, and because the long diameter of the fetal head is more frequent in the right oblique diameter of the pelvis subjecting the ureter on the right side to more pressure. It has also been asserted that right handed women sleep more upon the right side, and this would subject the right ureter to more pressure from the heavy uterus.

There are two forms of pyelitis in pregnancy; the one occuring in the early months due to an infection ascending from the bladder and usually of gonorrhoeal origin; the other occurs in the later months, the infection is through the blood and is us[illegible]due to the colon bacillus. This paper will deal mainly with this second class of cases.

*Etiology.*—The predisposing causes are: Lowered vitality of the patient; poor hygienic surroundings; alcoholic or venereal excesses; traumatism; calculi in the kidney or ureter. Frequently a history of some preceding gastro-intestinal disturbance can be obtained—this is interesting from the fact that the colon bacillus is the germ usually present in these cases. Any pelvic deformity or peri-uterine lesion may also be a predisposing factor.

The exciting cause is usually the colon bacillus. We have a "locus minoris resistentia" in the congested and dilated ureter and pelvis and we have an excellent culture medium in the stagnant urine. The mode of infection is through the blood, or hoematogenous. The infection is descending and not ascending, for if a cystitis developes it is secondary and not primary.

These attacks usually come on in the second half of pregnancy, the majority of the cases reported being in the fifth month or later.

This is because the pressure of the uterus upon the ureters is not so marked until the uterus has risen out of the pelvis.

One would suppose that it would be more frequent in primiparae on account of the greater pressure of their abdominal walls, but reports vary; one series of cases showed ten primiparae to four multiparae, another thirteen to eighteen; another seven to three; making a total of thirty primiparae to twenty-five multiparae. The age of the patients varied from 17 to 54 years.

*Pathology.*—Not many of these cases come to autopsy, but there has been found that the ureters and pelvis are irregularly dilated on one or both sides and they contain purulent urine. Microscopically the tissues are congested, ecchymotic, and show small round cell infiltration. The kidney may show inflammatory and suppurative changes, even to destruction of much of the kidney substance.

*Clinically.*—The cases are divided into the sudden or acute, and the insidious or mild. The former begin with an acute pain in one or both kidneys, there is a chill followed by a rapid rise in temperature up to 104 to 105 F., the pulse is rapid, and they may vomit. The pain may radiate down the ureters. There is frequent and painful micturition due to reflex spasm of the bladder; the urine is very cloudy and upon standing shows a large deposit of pus cells. The second or mild form comes on late in pregnancy, the onset is more gradual. They complain of some pain in the kidney region, usually the right; the temperature is not so high and they have less spasm of the bladder. These are the cases that are apt to be overlooked or improperly diagnosed.

*Symptoms.*—Pain is usually the first and most important.

There is a constant dull pain in the lumbar region of one or both sides, which become more acute by paroxysms and may radiate down the course of the ureters. The pain is due to the congestion and tension in the kidneys and is similar to nephritic colic. Complete occlusion of the ureter will make the pain more acute and it may be suddenly relieved, followed by a flow of a large quantity of purulent urine into the bladder.

*Temperature and Pulse.*—There is a mild apyretic form occuring late in pregnancy. The typical cases run a curve which resembles malaria; they have a chill followed by a rapid rise which continues a short time, they have headache, then the temperature falls and the patient perspires freely. The pulse goes up with the fever and may remain 100 or more after the temperature has fallen to normal. Digestive troubles usually precede the attack. There is often a history of diarrhoea or constipation, indigestion, anorexia and coated tongue.

There is a frequent desire to urinate, but only small quantities are passed. There may be sharp pain due to spasmodic contraction of the bladder. A true cystitis may develop late in the disease due to the infection travelling down the ureters. The total quantity of urine varies from day to day, but rarely do we get total anuria due to occlusion of both ureters.

*Physical Signs.*—The kidney of the affected side may be palpable and is always tender to pressure. In very advanced cases there may be a perinephritic oedema visible in the lumbar regions. Rarely can the ureter be palpated per vagina unless the inflammation has extended down to the bladder. The cystoscope will show purulent urine coming from the affected side.

*The Urine.*—The specific gravity is apt to be above normal; it is cloudy, containing much pus, some mucus and sometimes blood. The reaction is acid unless the bladder becomes involved. The quantity of pus varies being sometimes almost absent due to retention, then coming in large quantities due to the obstruction giving away.

An albumen reaction can be obtained due to the quantity of pus present or there may be a coexisting nephritis. Microscopically we find numerous pus cells, epithelial cells, few red cells, and bacteria, usually bacilli.

The general condition of the patients remains good in the mild cases, but in the severe cases there is more exhaustion, and if the trouble is bilateral it may cause grave anxiety.

The progress of the cases varies. The mild ones toward the end of pregnancy may show only pyuria with slight pain and general malaise, and this will all disappear with delivery. The acute cases may subside in eight to fifteen days and the trouble disappear; or the trouble may recur later in pregnancy; again the trouble may persist for some weeks and be terminated by pregnancy; or it may continue into the puerperium. It is not often that the condition goes on to suppuration of the kidneys, but it may do so and necessitate early delivery or operation upon the kidney. The trouble may come back in subsequent pregnancies, so patients should be informed of this possibility. Cragin advises that these cases should not be allowed to become pregnant again for at least one year after all traces of pus have left the urine.

*Diagnosis.*—There are numerous troubles which this condition may simulate. Cases have been mistaken for appendicitis on account of the pain in the side, fever and rapid pulse. Cases may also resemble typhoid or grippe. The chill and rapid rise of temperature may be mistaken for malaria. It has also to be differentiated from nephritic and hepatic colic, floating kidney, and salpingitis.

The marked pyuria is the chief diagnostic sign—this also emphasizes the necessity of careful urinalysis in all pregnant women. If the pyelitis occur during the puerperium it must be differentiated from intra uterine infection. It may cause grave alarm unless the true condition is recognized.

*Prognosis.*—The majority of these cases will recover under appropriate treatment in from one to six weeks. A few will abort or be delivered prematurely on account of the mother's condition. In severe cases there may be a prolonged labor due to the weakened condition of the mother—she is also more prone to postpartum complications. The prognosis in regard to the child is also good in the mild cases, but in the severe ones they are apt to be premature and weak.

*Treatment.*—The prophyllactic treatment consists in the proper hygienic measures during pregnancy, seeing that the patients have a proper amount of rest and avoid exposure. The digestive system should be kept in the best possible condition. Careful urinalyses should be made at short intervals.

The curative treatment consists in putting the patient to bed, having her lie upon the sound side to relieve the pressure upon the affected side. The diet should consist of fluids only. Large quantities of water should be taken to flush out the kidneys. A urinary antiseptic should be used and hexamethyltetramine (urotropin) grains five every four hours has given most excellent results, Salol in like doses may be added. The bowels should be kept open with saline laxatives.

Cystoscopy with ureteral catheterization and irrigation has been suggested, but as the majority of the cases recover without it and it would be very uncomfortable for the patients it has not been generally adopted.

In the severe cases which do not subside under the above treatment, we have to consider more active measures. If the child is viable and the mother is losing ground and there is fear of abscess in the kidney, the child should be delivered. If the inflammation should continue after the delivery of the child and an abscess form in the kidney a surgical operation must be performed. Nephrotomy will cure some cases, if not a nephrectomy will be necessary. Some authors claim that a primary nephrectomy gives better results than the nephrotomy with drain.

*Conclusion.*—There are two forms of pyelitis in pregnancy; one occurring in the earlier months and associated with cystitis, is an ascending infection usually of gonorrhoeal origin; the other form occurs in the latter half of pregnancy, is due to pressure on the ureters, stagnation of the urine, and infection with the colon bacillus.

The diagnosis of this condition is frequently confounded with other abdominal conditions or malaria. The pyuria is the chief diagnostic sign.

The cases which are promptly recognized and properly treated usually recover. Those which are overlooked or neglected may go on to abscess formation and destruction of the kidney.

BIBLIOGRAPHY:

Chamberlain: American Journal of Obstetrics. April, 1877.

Le Brigand: Revue Pratique d'Obstetrique et de Pediatrie. XIV, 1907.

Legneau: Annales des Maladies G. U. XXII, No. 19.

Cumston: New York Medical Journal, 1902. I, page 1123.
American Journal of the Medical Sciences.

Cathala: Paris Thesis, 1904.

Marteville: Paris Thesis, 1905.

Cragin: N. Y. Medical Record, July 16, 1904.
Surgery, Gyneacology and Obstetrics, Chicago, May, 1906.

Price: Chicago Medical Recorder, June 15, 1904.

Rochard: Presse Medicale, No. 92, 1904.

Spalding: American Journal of Obstetrics, February, 1905.

Opitz: Zeitschrift fur Geburtshulf und Gynaekologie, XLIV.

Ruppaner: Munchener Medicinischer Wochenschrift, LIII, No. 6.

Swift, etc.: Boston Medical and Surgical Journal, February 21, 1907.

Meek: American Journal of Ostetrics, February, 1907.

Reed: Surgery, Gynaecology and Obstetrics, February, 1907.

